# Introductory Lecture, to the Dental Class of 1858–9

**Published:** 1858-12

**Authors:** C. B. Chapman


### INTRODUCTORY LECTURE,

To the Dental Class of 1858-9.

BY PROF. C. B. CHAPMAN.

It was my aim, in my first introductory lecture in this institution, to present a narrative sketch of educational systems in the earliest historic centuries. My present object will be to glance at the history of the earliest educational establishments in the United States,—especially in the New England colonies,—reserving the narrative of others to another occasion. Our institutions are all comparatively modern. Still, in tracing the rise and progress of some of those which were first established, great changes will be found to have been wrought in them. Our country possesses unusual advantages in its historic treasures, in the fact that all which has been enacted upon its domains may be the subject of historic record, save, perhaps, that mysterious race who were the builders of those mounds of unknown origin which abound in different parts of our country.

The history of American colleges has been more faithfully written, than that of any other department of improvement. The scholor and the citizen may well and justly point, with laudable pride, to the inestimable value which was placed upon education in its various departments, by the care-worn occupants of the American colonies. It is in the history of the rise and improvement of American colleges, that we may discover the key to the impulse which led that gallant band of pioneers to forsake their native shores, and to seek an unoccupied country, where they could enjoy the sweets of civil and religious liberty. They were a feeble band; but they were conscious that if their posterity should attain that for which they had exposed themselves upon the mighty deep,—if they were destined to become an honorable and powerful nation,—it must be through their substantial intelligence; for they had been schooled in the nursery where the lesson was faithfully inculcated, that "knowledge is power." They believed that the most valuable legacy which they could transmit to their sons and daughters, would be, to bestow upon them well-organized institutions, in which a sufficient measure of education could be acquired to fit them to occupy honorable and useful positions in the various walks of life.

The early American colonists were men who understood and deeply felt the importance of education; and in pursuing their early plans, they founded a school or college in Newtown, in the vicinity of Boston, in 1636, which was but six years after the settlement of Boston. It would seem that this place gave a name to the institution; but, two years after, it was changed to Cambridge, in honor of the place where the principal men in the colony had received their education.

John Harvard, who gave a name to the institution when it assumed the character of a university, was educated at Emanuel College, in Cambridge, England, and was first settled as a minister in that country. He came to America in 1637, and was made a freeman in the colony before the close of that year. He preached a short time in Charlestown; but, being a victim to consumption, died before the close of 1638, leaving, by his will, one-half of his estate, which half consisted of £779 19*s.* and 2*d.*, for the erection of a College, as well as the whole of his library, which amounted to 320 volumes. A very brief memorial of Mr. Harvard has been preserved, from which it is inferred that he left a widow and an heir who was not his son.

The first name that can be found on the college records, as connected with the department of instruction, is that of Nathaniel Eaton, who assumed the management of the institution in 1637, in the double capacity of instructor and agent for receiving donations for the erection of a college building. Two years after, the General Court granted him 500 acres of land, on condition that he should continue in the employment for life. In this arrangement, however, the confidence of the managers was misplaced; for he soon proved to be a tyrant, as he grossly ill-treated the students by keeping them on scanty and improper food, and, finally, beat his usher (Nathaniel Briscoe) in an inhuman manner; for which act, he was fined 100 mark (£66), and dismissed from his position. Soon after this, he went to England, obtained a living in the established church, and became a violent persecutor of non-conformists; and, in 1640, ended his career in a debtor's prison.

In 1640, the General Court added to the income of the college, by granting the revenue of the ferry between Cambridge and Boston.

The most notable incident in the early history of this college, was its agency in the introduction of printing into the American colonies. The first printing press which was established north of Mexico, and the first and only one for many years in British North America, was at Cambridge, and as an appendage to Harvard College. This press was conferred through the agency of Rev. Joseph Glover, of England, who died on his voyage to this country; but the vessel having on board the press, and a person named Dage who had been employed by Mr. Glover for the purpose of conducting the printing, arrived in the autumn of 1638.

By direction of the magistrates and elders, Mr. Dage immediately set up the press at Cambridge; and, in the first month of 1639, commenced printing.

The first work from the American press, was the freeman oath; the next was Pierce's Almanac; and the third was the Psalms newly turned into meter. The first two were printed in 1639; and the third, of which there is a copy in the University library, was printed the next year. This was the first work which rose to the dignity of a book from the American Press.

The second president of Harvard College, was Rev. Henry Dunster; to which place he was elected, in 1640. The school, at that early period, took a high rank,—adopting all the better qualities of the English universities, and rejecting the vain pomp which so extensively existed in them.

The first commencement was held in 1642, on the second year of President Dunster's administration; at which, nine persons received the Bachelor's degree, which were the first degrees conferred in the British American colonies. President Dunster held his office in the college till 1656, which was fourteen years from the time of his appointment, with much credit to himself and great benefit to the institution. He withdrew, on account of some differences in theological belief from those adopted by the governors of the college, which were such as would not have induced such separation in one of less honor; but his peculiar ingenuousness led him to frankly declare his principles, and himself sanction and urge the separation, while he ever after cherished the most friendly relations with the managers of the institution. The property and patronage of the college very greatly improved during his administration, while his own salary was never more than £60, and, a part of the time, it did not exceed half that sum.

Some of the gifts and legacies to the college, at this early period, were of the most singular description.

In 1645, John Buckly, *Master of Arts*, and Matthew Day, *Stewart*, gave, for the use of the resident fellows, a garden containing an acre and a rod of ground, near the college, which was long called the fellows' and tutors' orchard. The record of some of the early gifts to Harvard College, will exhibit a view of the character and business habits of the people who were its early supporters. About 1640, "Israel Stoughton, of Dorchester, bequeathed to the college, towards the advancement of learning, 300 acres of land, about Marsh Brook." The same year, "John Newgate, of Boston, granted five pounds per annum forever, towards the maintainance of lawful, useful, and good literature therein, to be paid from the rents of his farm at Rumney Marsh." "John Coggan, of Boston, merchant, gave Harvard College, for the use of the president and fellows, so long as they may profess to teach the good knowledge of God's holy word and works, a parcel of land at Rumney Marsh, estimated at about seventy acres." The town of Cambridge gave 100 acres of land at Shawshin (now Billirace), to which President Dunster added 100 acres adjoining it.

Among these early gifts to this college, are some of a novel description. One person gave a number of sheep; another a quantity of cloth, valued at nine shillings; a third a pewter flaggon, worth ten shillings. A silver-lipped jug, a trencher, a salt cellar, completes a list which was faithfully recorded, with the donors' names.

The third president was Rev. Charles Chauncey, who was inaugurated on the 27th of November, 1654, and held the office for seventeen years. He was not only an efficient presiding officer and teacher, but a pattern of system and industry. He arose at four o'clock, and opened the exercises by expounding a chapter which had been read from the Old Testament in Hebrew, and, in the evening, a chapter from the New Testament was read from the Greek, and treated in the same way. During his administration, the press was actively employed, and was the object of much respect and attention from the colonists. The books which issued from this press (with the exception of almanacs), were mostly religious; and among the most important, were the publications of the apostle Elliot, in the Indian language. These were printed at the expense of a society in London, which was organized for the propagation of the gospel in New England. Some small treatises issued from this press, which were thought too liberal, and in 1662, the General Court saw fit to subject the press to the control of a commission, which consisted of Major General Gookin and Rev. Mr. Mitchel of Cambridge, who were appointed licensers. This order was rescinded, in 1663, and the press was restored to its former liberty, but for a few years only; for the fears of the government became again awakened, and it became more rigid than before. No press was allowed to be established, except at Cambridge, and every book must be issued under a license from the president of the college and three others, any two of whom could enforce the forfeiture of the press and employment.

In 1665, a house was erected at the expense of the London society, called the Indian Cottage, which was designed for the use of these people; but it was but little used by them.

It was fortunate, in an enterprise of this kind in the infancy of the colony, that so large a portion of its inhabitants were of one mind, and one faith; for all regarded the value of the college far above any other improvement to which they were asked to contribute, and all regarded it with an almost individual interest during the early years of its existence. The college must necessarily be under the supervision of scholars from the English universities, until persons could be reared from its own ranks, who were fitted, by education and discipline, to assume its management; but, since 1654, it has been under presidents who have been reared within its own walls. It seems almost incredible, how much was accomplished by these hardy sons of toil, when we remember that they had not only the work of subduing a wilderness,—of preparing the way for communication between the different colonies, by building bridges, making roads, building houses, preparing their clothing mostly within their own households; but, at the same time, the most vigilant care must be exercised in guarding against the stealthy attacks of their savage foes. A complete history of the progress of this institution, would be but a story of constant persevering toil, both on the part of teachers and their patrons; and a gradual improvement in the building, and apparatus, as well as the number of teachers and their more liberal support.

About the year 1683, and for a few succeeding years, the the college enjoyed the instructions of those remarkable men, the Mathers. Increase Mather was the son of a dissenting clergyman in England, who had been reared in all the rigor and religious faith of the puritans. The character of a noble mother seems to have been faithfully transmitted to the son. The character of his mother may be inferred from her expressed desire with regard to him, for she desired, above all things else, that he should possess grace and learning. This son was named, from a typical condition of the colonies, *Increase.*

It will be remembered that a son of Cotton Mather was an intimate friend of Dr. Franklin. In a letter to Samuel Mather, Franklin thus discourses, in relation to the influence of one of his father's essays, upon his youthful mind, and to it he attributed his desire to engage in useful pursuits. He says, "When I was a youth, I incidentally read a fragment of a book, entitled *Essays to do good.* It had been so little regarded by its former owner, that it was much mutilated, as several leaves were torn out; but the reading of what remained gave me such a turn of thinking, that led me to change all my plans and pursuits for life; for I have always set a greater value on the character of a doer of good, than on any other kind of reputation; and if I have been, (as you seem to think), a useful citizen, the public owes the benefits which I have conferred, to that book." Mrs. Mather is said to have inculcated in her son, the most rigid and systematic habits. She often put him in mind of the scripture lesson of wisdom, "Seest thou a man diligent in his business; he shall stand before kings, he shall not stand before mean men." When Mr. Mather was a candidate for graduation, an order was issued, by the governors, to detain his class most of a year longer than the usual time. This was highly resented by the class, seventeen of whom left the college, rather than comply; but his father, although not favorable to the measure, kept his son in college during the whole period, regarding it as most proper to give no countenance to any act which might seem like insubordination, although a degree of wrong was imposed.

It was not until 1674, that the General Court gave their consent to the establishment of a second press in the New England colonies.

To the honor of religion—pure and undefiled,—it may truly be said, that in every age it has ever been the foe to ignorance and superstition. Multitudes, in every age, may be referred to, eminent for the cultivation of human learning, and equally true to the principles of the Christian philosopher. Mr. Mather was one of this class. He was active in the organization of a philosophical society, which was in the habit of meeting once a fortnight, and of sending and receiving communications from the society of Leyden and the Royal Society of London.

In 1692, occurred one of the most trying incidents in the colonies. In this year, Charles II. called upon them to surrender their charter. Mr. Mather plainly and most decidedly opposed the measure, as a moral wrong. This he once did in a public meeting, called for the purpose of considering the matter, and so forcible and reasonable did his view of the question seem, that when he had concluded his speech, many were melted to tears. The responsibility of his course can only be appreciated, when we reflect that by this act he would surely incur the displeasure of the Crown and its adherents, and expose himself to prosecution for sedition. Mr. Mather was the first person in the colonies who was honored with the degree of Doctor of Divinity; and the ease with which this honor was bestowed, will be inferred, as seventy-nine years elapsed before the honor was again conferred,—the next recipient being Rev. Mr. Appleton, of Cambridge. In 1715, the clergymen in the province requested Mr. Mather to go to England, with an address from them as a body, to George I., on his accession to the throne. This honor he promptly refused, as he regarded the act as unnecessary and incompatible with their calling, and the position of the colonies.

About the first year of the last century, the college found a faithful and munificent benefactor in Mr. Thomas Hollis, of London, who first sent to his agents, in Boston, twelve casks of nails, and one of cutlery,—the avails of which was to be paid over to the agents of the college. This was soon augmented to £2,000, by small donations at short intervals. He next used his influence and means, in constituting a permanent fund, to be appropriated toward the support of ten indigent students, each of whom was to receive £10 annually. His last acts of munificence to this college were the endowment of a professorship of Divinity, a contribution toward the endowment of professorship of Mathematics and Natural Philosophy, as well as £2,000 for the purchase of apparatus and Library.

The first endowed professorship in Harvard College, and the first in this country, was the Divinity professorship which was founded by Mr. Hollis, in 1707, and which still bears his name, and Mr. Wigglesworth was the first person. During the same year, donations for the Library were received from Dr. Isaac Watts and others, his cotemporaries in Great Britain.

It would seem that corporeal punishment was inflicted here as late as 1674, for under that date is found the following entry, viz: "That Thomas Sargeant being convicted of speaking blasphemous words, shall be publicly whipped, before all the scholars. Which sentence was executed in the College Library. Rod for fool's back."

In the annals of events which preceded the foundation of *Yale College*, we find evidences of the peculiar magnanimity of the persons who were engaged in the establishment of this institution.

It was the original design of the colonists to found a college, at once, in each of the settlements; but wiser councils prevailed, which indicated the propriety of concentrating their means and influence upon one, until, from the extension of population, more should be demanded.

In the first book of records, after plans for the establishment of this college had been made and suspended, as the belief was expressed that they could not support an institution such as they wished, is found the following record under date of November 11th, 1644: "The proposition for the relief of poor scholars at the college in Cambridge was respectfully approved, and it was ordained that Joshua Atwater and Mr. Davis shall receive from every one in the plantation, whose heart is willing to contribute thereunto, one peck of wheat, or the value of it." In March, 1645, Mr. Atwater, the treasurer, informed the Court, that he had sent forty bushels of wheat for the college at Cambridge. In 1648, Samuel White was chosen collector of the college corn, for that year, in place of Anthony Johnson, deceased. It was also ordained, that the college should be called "the school of the church," and all churches were invited to contribute to its support. Ten ministers were nominated and agreed to by both ministers and people, who were to stand as trustees or undertakers, to found and govern a college. This was about the year 1699; and in 1700, they met at New Haven, and formed themselves into a society, which consisted of eleven ministers, including a rector, and agreed to found a college in the colony of Connecticut. This agreement was soon after carried into effect, in a manner peculiarly characteristic of the simplicity of the age. "Each member brought a number of books and presented them to the body, and laying them on the table, said these words—"I give these books for the founding of a college in this colony." The trustees then took formal possession of them, which, with a small addition from other sources, constituted about forty folios, and some money, which, in the language of President Clapp, "laid a good foundation."

The original charter bears date of 1701, when the General Court granted £120, or £60 sterling, annually, until otherwise ordered. The first commencement was held at Saybrook, on the 13th of September, 1702, at which time, six students received the Bachelor's degree. These students had been instructed by the different trustees; and the rector, Mr. Pierson, continued to reside at Killingworth, where he died, in 1707.

The extent to which these classes were disposed, to conform to the exigencies of the times, will be inferred, when we read that the senior class was removed to Milford after the death of Mr. Pierson, in order to be instructed by Rev. Samuel Andrew, who was chosen Rector *pro tem.*, to serve until one could be obtained who would reside at the collegiate school. The other students were removed to Saybrook, and placed under the care of two tutors, so that the students who belonged to different classes in that college, were only assembled at stated times, for examinations and at commencements.

The college continued, in this obscure condition, at Saybrook, until 1713, when a new impulse was given to the enterprise by several considerable donations of books. In

1714, a still more liberal addition was made to the library, through the influence of Jeremiah Drummond, of Boston, then the colonial agent in London. He sent eight hundred volumes, one hundred and twenty of which were at his own cost. Among the donors of this collection, were associated some honorable and worthy names, including Sir Isaac Newton. The gifts of books, for that year, was completed by one from Governor Yale, of about forty very valuable volumes. The number of graduates, from 1702 to 1713, was forty-six, of which number, thirty-four became ministers.

A home for the college was not yet established, and although an impulse was given to the enterprise, by the above donations, an impediment to its immediate progress was presented, by a rivalry of several towns, for the place where the college should be fixed. Although *Saybrook* was not a favorable place, its claims were strongly urged, together with *Weathersfield*, *Hartford*, and *New Haven*. Those who advocated its location at each place, were often influenced by circumstances of pecuniary or personal accommodation. These manifestations of dissatisfaction having become apparent among the students, the trustees met at Saybrook, in 1716, and gave permission to such as were discontented to go to other places for instruction until the next commencement. Pending these manifestations of dissatisfaction, the people in the several parts of the colony, adopted the very proper plan, of subscribing for the erection of a college, such sums as they were disposed to bestow to given places. When these subscriptions were closed, it was found that Saybrook and New Haven had the largest sums, when others were induced to transfer their subscriptions to one of these places, which they did in sums which accorded with their comparative estimate of the places. When this plan was completed, New Haven was found to have £700 and Saybrook £500 sterling. At a commencement, held at Saybrook, the 12th of September, 1716, the trustees agreed to meet at New Haven the following month, when they voted,

"that, considering the diffiulties of continuing the collegiate school at Saybrook, and as New Haven is a very convenient place, and as it has received the most liberal donations, the trustees agreed to remove the school to that place." This measure met with a strong opposition from places which had competed, and the contest was so sharp that the minority of the board of trustees, repeatedly remonstrated with the general assembly on the subject.

In 1718, a new direction was given to the question, by a large donation, in books and merchandise, which was bestowed by the Honorable Eliphalet Yale, of London, who was then Governor of the East India Company.

In September, 1718, a splendid commencement was held at New Haven, when the name of the college was formally changed to YALE, in honor of the munificent donor of that name. The opposition, however, was not quite silenced, for a sort of commencement was, at the same time, held at Weathersfield, where five diplomas were granted. In order to conciliate these persons, the evidences of graduation was granted them from Yale. The last manifestation of opposition was shown when the library and other effects of the corporation were removed from Saybrook; for so great was the feeling there, that although it was done under the direction of the sheriff with legal process, they were obstructed by a mob, and some hundred and fifty valuable books and manuscripts were taken by unknown persons, and never recovered.

The incidents which transpired during the next half century, in the history of this college, were not such as to demand more than a passing notice. The presiding officers during this time, were as follows:—Timothy Cutter, L. L. D., was elected Rector, 1719; Elisha Williams, 1726; Thomas Clapp, 1740.

President Clapp was ardently devoted, not only to his calling as a teacher, but to various improvements in the mechanical and useful arts. He is said to have constructed the first orrery in America. When a drill, being out of repair, could not be mended by any mechanic in the town, he undertook and succeeded in the task, as well as added some new value to the instrument.

President Clapp was succeeded by Nepthali Daggett, who was installed in 1766, and resigned his office in 1777, but retained his place as professor of Divinity, to which he had been chosen when but twenty-eight years old. The character of President Daggett may be inferred, when we are informed that he was chosen at that early age, by a body of men who were distinguished alike for their classical as well as their theological learning. His presidency was at a time, as the dates will indicate, when the country was agitated by the incidents which preceded the war of the Revolution, and his voice, in common with that of the New England clergy, was used with good effect in stimulating his countrymen to the defence of their homes. On the 5th day of July, 1779, about 2,000 British soldiers, whose only object seemed to be plunder and wanton acts of cruelty, entered New Haven. They were only resisted by a few feeble militia and citizens, at the head of whom was the venerable professor of Divinity, who nobly used his musket until compelled to surrender it. When taken prisoner, he was cruelly treated, which was probably the cause of bringing him much sooner to the termination of his valuable life.

The incidents connected with the Revolution were not calculated to favor those acts and employments which would directly promote the foundation and patronage of a college. Still, the people of the colonies were deeply impressed with the importance of maintaining that civil and religious liberty, to secure which they had left their homes across the Atlantic and taken up their abode in the wilderness of the western hemisphere. They had sacrificed too much in order to secure these privileges, to be induced to lightly yield that which to them was dearer than life itself; for it was not for themselves, but for their posterity, they had endured so much.

After seven years of almost entire suspension of the exercises of the college, President Stiles resumed its government, and new life seemed to have been infused into it, immediately after peace and liberty had been established. President Stiles died in (12th May) 1795, at which time, 2,372 students had been educated here. Dr. Timothy Dwight, who succeeded, represented, that President Stiles was probably the most learned man in America, and exceeded by few in the world. Dr. Dwight was not only a finished scholar, and efficient president, but an ardent patriot, as may be inferred from his authorship of that patriotic song—

"Columbia, Columbia, to glory arise!
The Queen of the world, the Child of the skies,"

which was so universally taught by mothers, to the children of the last generation.

At this time, there was but one professor in the institution, who, with the president and three tutors, conducted the department of instruction in the college.

Tutorships were mostly accepted on account of the temporary support, and perhaps some literary advantages, which they afforded, and were consequently quite temporary.

Dr. Dwight applied his vigorous powers to the enlargement of collegiate advantages. In 1803, Rev. Jeremiah Day was chosen to the Chair of Mathematics and Natural Philosophy, a professorship which had been founded by the General Court, in 1770, and occupied by Mr. Meigs, who had just accepted the presidency of the University of Georgia.

In 1804, Benjamin Silliman was chosen professor of Chemistry and Mineralogy, and immediately visited Europe in order to improve himself in his department.

So much had the institutions of this country been under the influence of venerable follies, that, until the time of Dr. Dwight, the printed catalogue of students was arranged according to respectability of parentage, and freshmen were compelled to perform menial offices for the two higher classes. This absurd and unjust rule was not abolished until 1804.

The first degree of M. D., from this institution, was conferred, in 1723, upon Daniel Turner, of London, who had bestowed a liberal donation of books upon the college.

A Law department was established, in 1801, and Elijah Goodrich appointed instructor.

In 1810, a medical society was incorporated, which resulted in the establishment of a Medical department, and the first course of lectures was delivered in 1813, to thirty-six students. This arrangement was mostly accomplished through the exertions of Dr. Nathan Smith, who was a striking example of what one individual of untiring industry may accomplish. In 1829, the Medical department was made still more complete, by the appointment of Drs. Hubbard and Tully. The latter held his place till 1848, when he retired to Springfield, Massachusetts, in order to engage in the publication of his American Medical Botany, in which employment, although at an advanced age, he is still engaged.

I have thus rapidly glanced at the prominent incidents connected with the origin and development of these two leading institutions of our country. Time will not admit of a satisfactory reference to others,—the history of which would be no less interesting, although not so directly connected with the early characteristics of the American colonists.

I have chosen to give you this narrative sketch, on this occasion, for the purpose of presenting the fact, that the most important, because the most useful, enterprises in which we may engage, and to which our most active energies ought to be directed, are seldom brilliant or attractive in their beginning, because every thing useful involves labor and sacrifice.

Our ancestors seemed to regard the support of their institutions of learning as demanding their first care, after the supply of their daily natural wants; and to this their solicitude are we indebted for the position we hold, as a nation, among the many enlightened nations of the globe.

Their varied toils, we are not called to endure. They have set us a noble example,—and shall we prove worthy of our more than noble ancestors?

Institutions of learning are no less needed, because so abundant and diversified in their character; and every good citizen, of whatever age or condition, is bound by a principle of honor, to foster and support, by patronage and means, such as are in their midst.

Institutions for instruction in the dental art and science are of recent origin.

The idea of the necessity of such institutions, seems to have been first cherished by your worthy teacher of the Institutes of Dental Science, Prof. Taylor, who proposed to Dr. Chapin A. Harris, member of the Baltimore College, to engage in such an enterprise. After various conferences, in which, plans were somewhat perfected, it was found that their individual preferences, in choice of location, could not be gratified by a union in the enterprise, which very properly led to the almost simultaneous establishment of the two colleges. The history of these institutions, so far as individual sacrifice is concerned, is but faithfully written in the narrative I have given you of other colleges. We can not claim from the general public that patronage which has been so liberally bestowed upon primary and general literary institutions. Ours is a special department of education, and in such the general public will take but little interest. From the midst of our own ranks, must we mostly look for the aid and comfort which we must have, in order to render our labors as useful as they ought to be; and, so far as you are concerned, shall we be disappointed? Will you, when your term of study with us has expired, and as you go forth to reap some of the benefits we may have conferred upon you, fail to regard your Alma Mater as an object of solicitude and care?

